# Prevalence of clinically apparent hypertrophic cardiomyopathy in Germany—An analysis of over 5 million patients

**DOI:** 10.1371/journal.pone.0196612

**Published:** 2018-05-03

**Authors:** Daniela Husser, Laura Ueberham, Josephine Jacob, Denise Heuer, Steffi Riedel-Heller, Jochen Walker, Gerhard Hindricks, Andreas Bollmann

**Affiliations:** 1 Department of Electrophysiology, Heart Center Leipzig and Leipzig Heart Institute, Leipzig, Germany; 2 Institute for Applied Health Research (InGef), Berlin, Germany; 3 Institute of Social Medicine, Occupational Health and Public Health, Leipzig University, Leipzig, Germany; Medical University Innsbruck, AUSTRIA

## Abstract

**Background:**

Hypertrophic cardiomyopathy (HCM) is the most common inherited heart disease. Reported prevalence rates vary substantially between 1:500 (0.2%) and 1:3,000 (0.03%), which may be attributed to different study designs and population characteristics.

Prevalence data for Germany is not available. Consequently, this study aimed (1) to quantify age- and gender-specific clinically diagnosed HCM prevalence in Germany based on the analysis of health care claims data of > 5 million insurants in 2015, and (2) to analyze temporal prevalence trends from 2011 to 2015.

**Methods:**

Data were extracted from the InGef (Insitute for Applied Health Research) database, which is an anonymized healthcare claims database with longitudinal data from patients insured in one of approximately 70 German social health insurances (SHIs). Patients were classified as HCM prevalent, if they had at least one verified ambulatory or one hospital main- or secondary discharge diagnosis of HCM (I42.1 or I42.2).

**Results:**

In 2015, HCM was prevalent in 4,000 out of 5,490,810 patients (0.07%; 1:1,372). HCM prevalence increased gradually with age from 7.4/100,000 persons (95% CI 5.2–10.1) in 0–9 years old to 298.7/100,000 persons (95% CI 276.4–322.4) in patients > 80 years. In all age categories, men had a numerically higher prevalence than women with significant differences in patients > 30 years. There was a gradual annual prevalence increase from 75.8 (95% CI 75.2–76.4) in 2011 to 84.2 (95% CI 83.5–84.8) in 2015 per 100,000 persons.

**Conclusions:**

Overall, prevalence of clinically diagnosed HCM in Germany is lower than in systematic population studies based on echocardiographic diagnosis. Prevalence increased with advancing age and showed a constant yearly rise. Those observations may improve our understanding of the burden of this genetic heart disease on the health care system in Germany, increase the diagnostic awareness among clinicians and shape future screening and management strategies.

## Introduction

Hypertrophic cardiomyopathy (HCM) is the most common inherited heart disease that is defined by the presence of increased left ventricular (LV) wall thickness that is not solely explained by abnormal loading conditions. It is a clinically and genetically heterogeneous disorder and an important cause of sudden death and heart failure. Early diagnosis of HCM is important for providing appropriate treatment and prevention strategies but also for initiating clinical and genetic surveillance and counseling of family members [1].

HCM prevalence is commonly reported as 1 in 500 persons (0.2%) which was originally based on the CARDIA (Coronary Artery Risk Development in Young Adults) cohort study that used standard echocardiography in 4,111 unrelated people 23 to 35 years of age [2]. Importantly, subjects were randomly selected from the general population in community-based urban centers in which a substantial proportion of affected subjects has not come to clinical recognition. This prevalence estimate was subsequently corroborated by several studies with diverse study designs and cohort characteristics including different age groups and ethnicities [3–5]. This prevalence is in stark contrast to a recent analysis of U.S. claims data that found a prevalence of clinically diagnosed HCM in approximately 1:3,000 (0.03%) [6].

Comparable contemporary data from Germany are lacking. Therefore, the aims of this study were (1) to quantify age- and gender-specific clinically apparent HCM prevalence in Germany based on the analysis of claims-based data of > 5 million patients in 2015, and (2) to analyze temporal prevalence trends between 2011 and 2015.

## Patients and methods

### Data source

This study used the InGef (Institute for Applied Health Research Berlin) database which is an anonymized healthcare claims database with longitudinal data over a look-back period of up to six years from approximately 6.7 million Germans insured in one of approximately 70 German social health insurances (SHIs) currently contributing data to the database (mainly company or guild health insurances). Claims data are transferred directly from the healthcare providers to a specialized data center owned by the SHIs, which provides data warehouse and information technology services. In the data center, data are anonymized before entering the InGef database. Data are anonymized with respect to individual insurant, healthcare providers (e.g. physicians, practices, hospitals and pharmacies) and the respective SHI. The estimated delay of data being available in the database is approximately 3 to 9 months (e.g. healthcare data until 31 December 2016 can be expected to be available in the database at September of 2017 at the latest). The most important data elements included in the database are demographic information (including the date of death if applicable); ambulatory services and ambulatory diagnoses; hospitalization information including date of admission and discharge, diagnoses and procedures.

Diagnoses were coded via the International Statistical Classification of Diseases and Related Health Problems (ICD-10-GM [German Modification]).

### Study population

The InGef research database was used to conduct yearly retrospective cohort studies to estimate the population-based HCM prevalence per 100,000 persons for each year between 2011 and 2015.

In order to estimate the HCM prevalence, yearly population-based cohorts were created between 2011 and 2015. Subjects were eligible to enter the cohort in the respective year, if they fulfilled all of the following criteria in the respective year: (1) continuous enrolment in the SHI in the respective year or from birth onwards or until death (base population at risk = denominator); (2) no concomitant diagnosis of HCM phenocopies (mitochondrial disease (G31.81), Danon disease (E74.0x), Friedreich’s ataxia (G11.3x), Leopard syndrome (Q87.8x), Noonan syndrome (Q87.1x), Anderson–Fabry disease (E75.2x), Amyloidosis (E85x). Patients with at least one ambulatory verified or a hospital main- or secondary discharge diagnosis of HCM (I42.1 or I42.2) between 01.01.and 31.12. of the respective year were considered HCM cases.

Cohort entry was the 1st January of the respective study year, if all inclusion criteria were fulfilled, or the date of birth for newborns. Cohort exit was defined as the occurrence of HCM, death, or the 31st December of the respective study year, whichever occurred first.

## Statistical analyses

The HCM prevalence per 100,000 persons was calculated stratified by sex and age-group for each year between 2011 and 2015 by dividing the absolute number of HCM cases by the total number of persons in the base cohort.

In order to be able to report prevalence estimates representative for the German population the crude rates were directly standardized to the age and gender distribution of the German population in the respective calendar year. In direct standardization, a standard population is used to eliminate effects of any differences in age and gender between two or more populations being compared, in this case between the population in the base population in the InGef database and the German population. Direct standardization entails the calculation of weighted averages of the stratum-specific rates in the study population, using the corresponding number of subjects in each stratum of the standard population as weights, in this case the German population according to Destatis (https://www-genesis.destatis.de/genesis/online). Thereto direct adjustment weights are calculated by dividing the number of patients in each age and gender stratum in the German population by the number of patients in the same age and gender stratum in the InGef database. Subsequently all patients in the InGef database were assigned their age and gender specific weight. The sum of all weights across all patients in the study population corresponds to the overall number of patients with HCM in the German population.

Frequency of comorbidities and the Charlson index [7] were analyzed.

Continuous variables are presented as mean ± one standard deviation and categorical variables as frequencies. For descriptive and comparative purposes, 95% confidence intervals are provided assuming a Poisson distribution. Statistical analysis was performed using SAS version 9.4.

## Results

**Fig 1:**
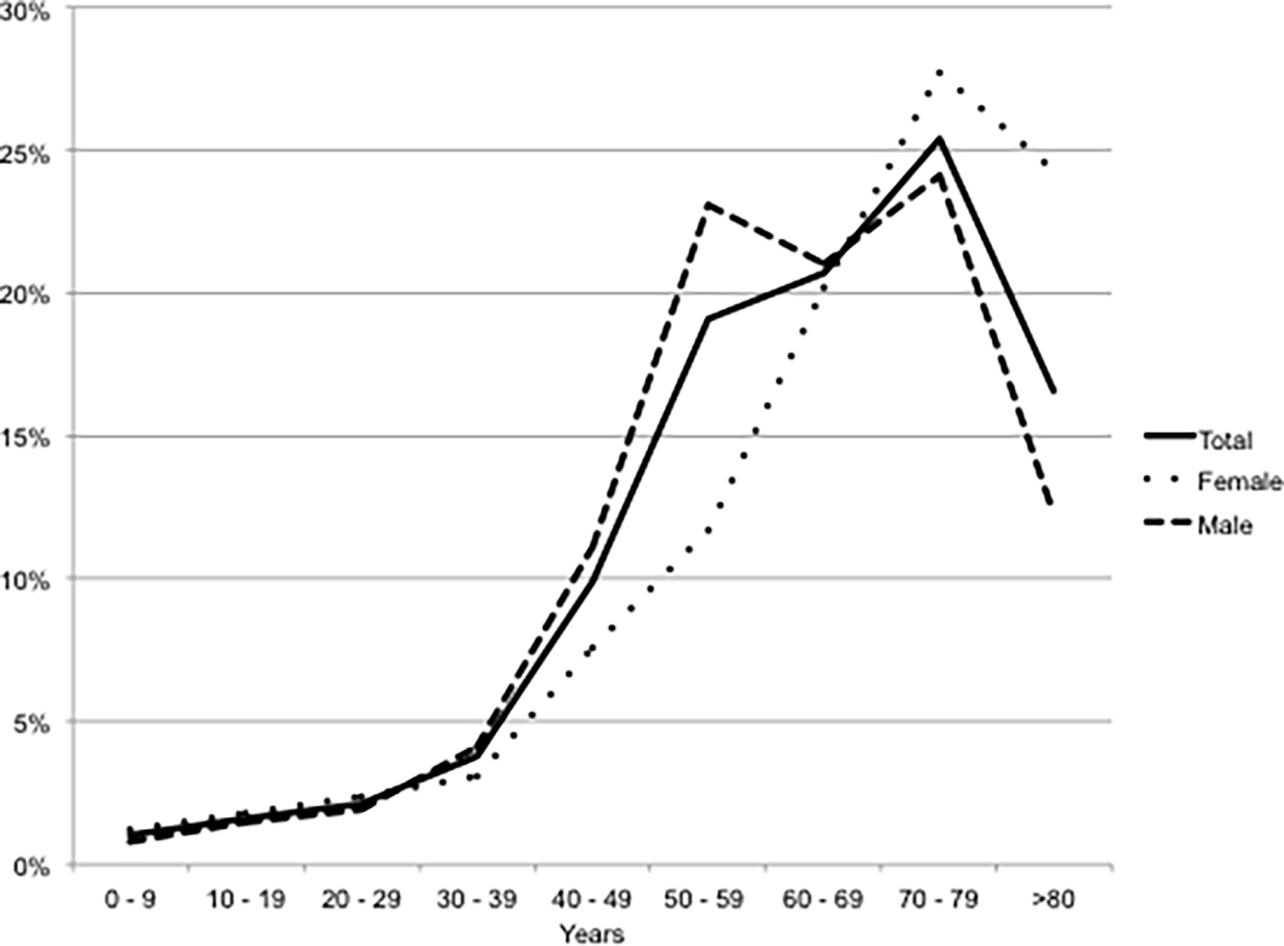
Gender-stratified age distribution of the HCM cohort.

In 2015, HCM was prevalent in 4,000 out of 5,490,810 patients (0.07%; 1:1,372). The average age of these patients was 63±17 years (median 66 years), and 2,586 (65%) were male. Female patients were significantly older than males (66±18 vs. 61±16 years, p<0.0001). The gender-stratified age distribution of this cohort is depicted in Fig 1.

Patients had a Charlson comorbidity index of 2.96±2.63 with the 10 most common co-morbidities summarized in Table 1.

**Table 1:** Co-morbidities with corresponding ICD-10 codes (in %) of HCM patients.

Essential (primary) hypertension	I10/I11	80.7
Disorders of lipoprotein metabolism and other lipidemias	E78	53.3
Dorsalgia	M54	38.5
Chronic ischemic heart disease	I25	38.1
Disorders of refraction and accommodation	H52	34.4
Heart failure	I50	34.3
Overweight and obesity	E66	28.1
Type 2 diabetes mellitus	E11	26.7
Cardiac arrhythmias^a^	I49	26.6
Nonrheumatic mitral valve disorders	I34	25.2

^a^ventricular arrhythmias, premature atrial, nodal or ventricular complexes, sick sinus syndrome

The prevalence of HCM increased successively with age from 7.4/100,000 persons (95% CI 5.2–10.1) between 0 and 9 years to 298.7/100,000 persons (95% CI 276.4–322.4) in patients > 80 years (Table 2). In all age categories, men had a numerically higher prevalence than women with significant differences in patients > 30 years (Table 2).

**Table 2:** Age- and gender-specific HCM prevalence in 2015.

		Total			Female			Male	
Age category	Crude rate	Lower 95% CI	Upper 95% CI	Crude rate	Lower 95% CI	Upper 95% CI	Crude rate	Lower 95% CI	Upper 95% CI
0–9 years	7.4	5.2	10.1	6.8	3.9	10.8	7.9	4.9	12.1
10–19 years	11.2	8.7	14.3	9.2	6.0	13.6	13.1	9.3	17.9
20–29 years	12.5	10.0	15.5	10.7	7.4	14.9	14.3	10.5	18.9
30–39 years	20.6	17.4	24.1	11.6	8.4	15.5	30.3	24.8	36.6
40–49 years	43.1	39.0	47.6	23.0	18.8	27.8	64.0	56.8	71.8
50–59 years	83.2	77.4	89.4	36.6	31.3	42.6	128.8	118.6	139.5
60–69 years	149.9	139.8	160.4	103.4	91.8	116.2	195.9	179.8	213.1
70–79 years	254.9	239.4	271.0	200.0	180.7	220.8	307.9	284.2	333.1
80+ years	298.7	276.4	322.4	270.5	242.6	300.6	336.6	300.6	375.6
Total	72.8	70.6	75.1	51.4	48.8	54.2	94.4	90.8	98.1

**Fig 2:**
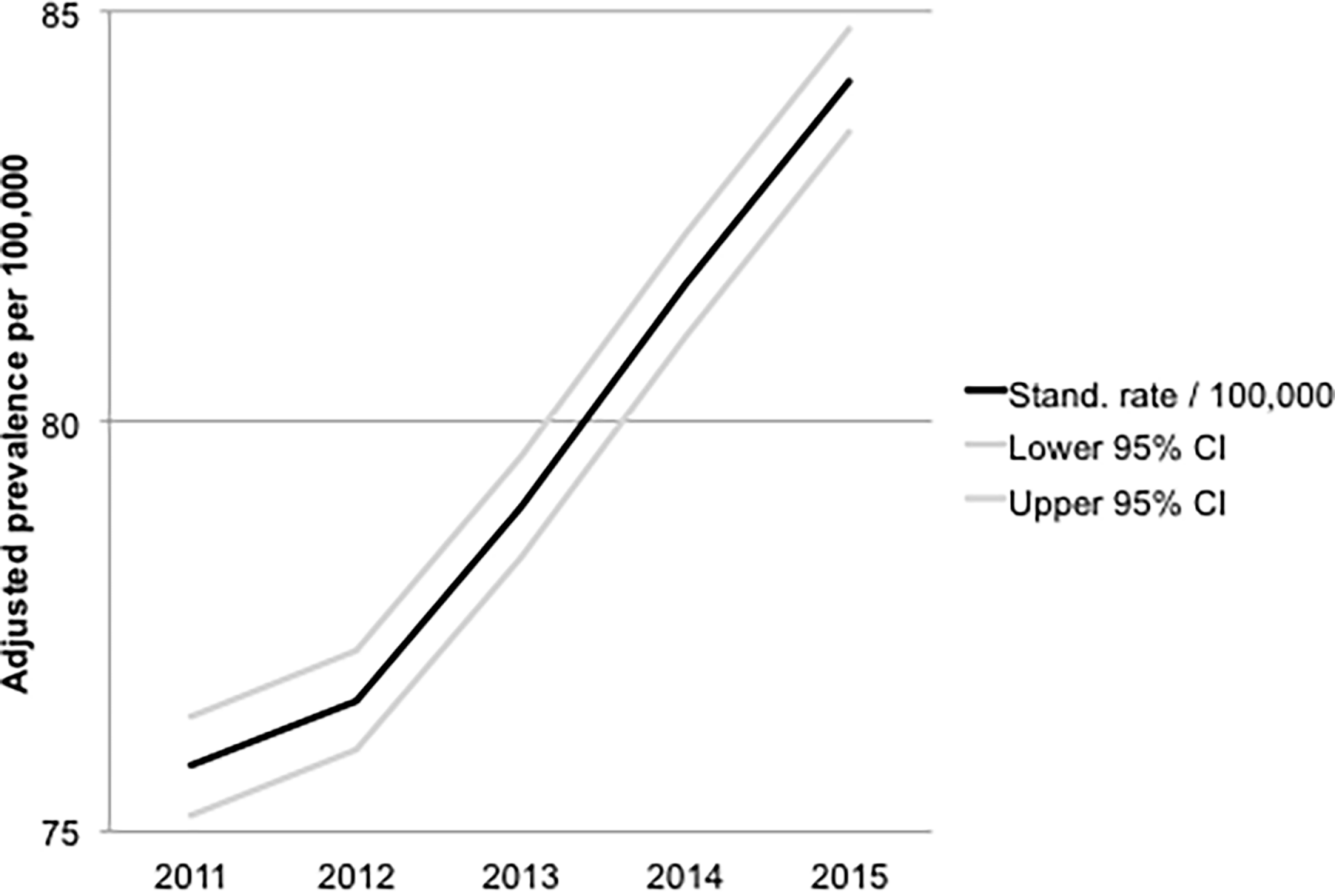
Temporal trends of HCM prevalence.

Temporal trends of age- and gender-standardized HCM prevalence are summarized in Fig 2. There was a gradual annual increase from 75.8/100,000 persons (95% CI 75.2–76.4) in 2011 to 84.2/100,000 persons (95% CI 83.5–84.8) in 2015.

## Discussion

### Main findings

This study is–to the best of our knowledge–the first to systematically explore HCM prevalence of clinically apparent cases in Germany. For this purpose, a claims database of more than 5 million patients was analyzed and revealed (1) a lower prevalence of diagnosed HCM than in cohort studies based on echocardiographic diagnosis; (2) gradual HCM prevalence increases with advancing age and (3) from the year 2011 to 2015.

### Comparison with previous studies

Several studies have established a HCM prevalence of about 0.2% (1:500 cases) in adults [2–5]. Importantly, those studies used population screening by echocardiography that also allowed detection of clinically silent cases. Consequently, it is not surprising that the number of clinically diagnosed cases was lower in U.S. claims data [6]. While in the U.S. study HCM was found in 0.03%, we detected clinically apparent cases in 0.07%, which has been considered to represent “the tip of the iceberg of the disease spectrum” [6]. This is also in accordance with a similar smaller study from Sweden that reported a prevalence of 0.04% [8]. The discrepancies become even more prominent when genetic population studies are being factored in. Based on these data, the minimal prevalence of HCM gene carriers could be estimated at 0.5% (1 in 200 people) [9]. Although not all gene carriers may develop clinical HCM, the high frequency of HCM-causing pathogenic mutations strongly suggests a prevalence exceeding that reported in echocardiographic screening studies and by far exceeding that found when conducting claims-based analysis.

Traditionally, HCM has been regarded as a disease of the young, but is nowadays identified with increasing frequency in older patients. For instance, 25% of HCM patients in a community-based cohort not subject to tertiary center referral bias were > 75 years [10] which is in agreement with our observations. The notion that HCM may remain clinically silent for long periods of time with symptoms and initial diagnosis deferred until late in life may at least in part be explained by age-dependent penetrance of disease causing mutations and the presence of additional clinical and genetic modifiers. For instance, sarcomere protein mutations such as mutations in cardiac myosin binding protein-C, troponin I, and α-cardiac myosin heavy chain have been shown to cause elderly-onset HCM. This mutation spectrum is strikingly different from that of familial, early-onset HCM [11]. The presence of an additional risk factor for LV hypertrophy has been identified as modulator for the phenotypic expression of HCM [12]. Interestingly, hypertension–which was also found in the majority of our HCM cases–has been associated with later HCM diagnosis [13].

While HCM is most frequently transmitted as an autosomal-dominant trait most studies have reported a male preponderance that was also found in our study. This observation remains largely unexplained but might also reflect the impact of genetic and hormonal modifiers [14]. In addition, hypertension as modulating factor is more prevalent in males which may result on more frequent echocardiographic examinations and subsequent HCM diagnosis.

Recent advances such as better understanding of the underlying molecular and genetic pathophysiology, implementation of contemporary family screening, more sensitive diagnostic cardiac imaging, better risk stratification with improved outcomes may also contribute to the increasing prevalence of this disease.

### Limitations

Limitations of this analysis include those inherent to claims database studies, such as the reliance on accurate coding and diagnosis. Consequently, results need to be interpreted cautiously. While in one study, accuracy of HCM coding was high [15], there were some misclassifications found in another study [8]. Since there is lack of clinical, ECG and imaging information database diagnoses could not be verified. Furthermore anonymized claims data cannot be combined with clinical records to confirm HCM diagnoses documented in claims data. In particular, the high prevalence of hypertension could have led to misdiagnosis, acting not only as modulator, but also as cause for secondary form of hypertrophy which would result in even lower overall HCM prevalence. Nevertheless, the discrepancy between claims data analysis revealing clinically diagnosed cases and prevalence studies based on outreach programs is striking.

Approximately 10% of the German population with private medical insurance is not covered in this database, which limits the extrapolation of the results to this population. Nevertheless, this database has been shown to be representative of the German population demographics and the directly standardized prevalence was calculated to account for any differences in age and gender distribution between the InGef database and the German population [16].

## Conclusions

Overall, prevalence of clinically diagnosed HCM in Germany is lower than in systematic population studies based on echocardiographic diagnosis. Prevalence rates increased with advancing age and showed a constant yearly rise. Those observations may improve our understanding of the burden of this genetic heart disease on the health care system in Germany, increase the diagnostic awareness among clinicians and shape future screening and management strategies.
